# Effects of Decreased Vitamin D and Accumulated Uremic Toxin on Human CYP3A4 Activity in Patients with End-Stage Renal Disease

**DOI:** 10.3390/toxins5081475

**Published:** 2013-08-19

**Authors:** Masayuki Tsujimoto, Yui Nagano, Satomi Hosoda, Asuka Shiraishi, Ayaka Miyoshi, Shima Hiraoka, Taku Furukubo, Satoshi Izumi, Tomoyuki Yamakawa, Tetsuya Minegaki, Kohshi Nishiguchi

**Affiliations:** 1 Department of Clinical Pharmacy, Faculty of Pharmaceutical Science, Kyoto Pharmaceutical University, Kyoto 607-8414, Japan; 2 Department of Pharmacy Service, Shirasagi Hospital, Osaka 546-0002, Japan; 3 Department of Medicine, Shirasagi Hospital, Osaka 546-0002, Japan

**Keywords:** CYP3A4, 1,25-dihydroxyvitamin D, uremic toxins, end-stage renal disease, vitamin D receptor (VDR)

## Abstract

In patients with end-stage renal disease, not only renal clearance but also hepatic clearance is known to be impaired. For instance, the concentration of erythromycin, a substrate of cytochrome P450 3A4 (CYP3A4), has been reported to be elevated in patients with end-stage renal disease. The purpose of this study is to elucidate the reason for the decrease in hepatic clearance in patients with end-stage renal disease. Deproteinized pooled sera were used to assess the effects of low-molecular-weight uremic toxins on CYP3A4 activity in human liver microsomes and human LS180 cells. Four uremic toxins (3-carboxy-4-methyl-5-propyl-2-furanpropanoic acid, hippuric acid, indole-3-acetic acid, and 3-indoxyl sulfate) present at high concentrations in uremic serum were also studied. Simultaneous treatment of uremic serum (less than 10%) or uremic toxins did not affect testosterone 6β-hydroxylation in human liver microsomes. On the other hand, pretreatment of each serum activates CYP3A4 in LS180 cells, and the increased CYP3A4 activity in uremic serum-treated cells was smaller than normal serum-treated cells. In addition, CYP3A4 and CYP24A1 mRNA levels also increased in LS180 cells exposed to normal serum, and this effect was reduced in uremic serum-treated cells and in cells exposed to uremic serum added to normal serum. Furthermore, addition of 1,25-dihydroxyvitamin D to uremic serum partially restored the serum effect on CYP3A4 expression. The present study suggests that the decrease of 1,25-dihydroxyvitamin D and the accumulation of uremic toxins contributed to the decreased hepatic clearance of CYP3A4 substrates in patients with end-stage renal disease.

## 1. Introduction

In clinical pharmacokinetics, drugs that are predominantly cleared by the kidney require dose adjustments in patients with end-stage renal disease (ESRD). In these patients, dose adjustment is often overlooked for drugs that are predominantly cleared by metabolism and/or transport. However, non-renal clearance of compounds requiring hepatic metabolic clearance is also known to be reduced in ESRD patients [1,2].

Cytochrome P450 (CYP) 3A4 is an important drug-metabolizing enzyme with many substrates, some of which are administered to patients with ESRD in clinical situations. However, the concentration of erythromycin, which is a CYP3A4 and hepatic uptake transporter, organic anion transporting polypeptide (OATP) substrate, has been reported to be elevated in ESRD patients [3]; uremic toxins 3-carboxy-4-methyl-5-propyl-2-furanpropanoic acid (CMPF) and 3-indoxyl sulfate inhibit erythromycin metabolism in rat hepatocytes [4]. Furthermore, we have previously reported that uremic serum and uremic toxins inhibited the metabolic clearance of losartan in human liver microsomes (HLMs) [5]. Therefore, the decreased non-renal clearance of erythromycin may reflect CYP3A4 dysfunction.

The expression of CYP3A4 is controlled by nuclear receptors such as pregnane X receptor (PXR) [6] and vitamin D receptor (VDR) [7]. 3-Indoxyl sulfate induces CYP1A1 mRNA expression by activation of the nuclear arylhydrocarbon receptor (AhR) [8]. Furthermore, hepatic Cyp3a2 mRNA and protein levels in rats with chronic renal failure are lower than the corresponding levels in normal rats [9]. Therefore, PXR and/or VDR may be inactivated by uremic toxins found at high concentrations in uremic serum.

The objective of this study is to elucidate the reason for the decreased non-renal clearance of CYP3A4 substrates in ESRD patients. The effects of uremic serum obtained from ESRD patients and uremic toxins on CYP3A4 activity in HLMs were investigated. In addition, CYP3A4 activity and expression were investigated in uremic toxin-treated LS180 cells, a human colon adenocarcinoma cell line that expresses PXR and VDR.

## 2. Results

### 2.1. Effect of Uremic Serum or Uremic Toxins on Testosterone 6β-Hydroxylation in HLMs

**Figure 1:**
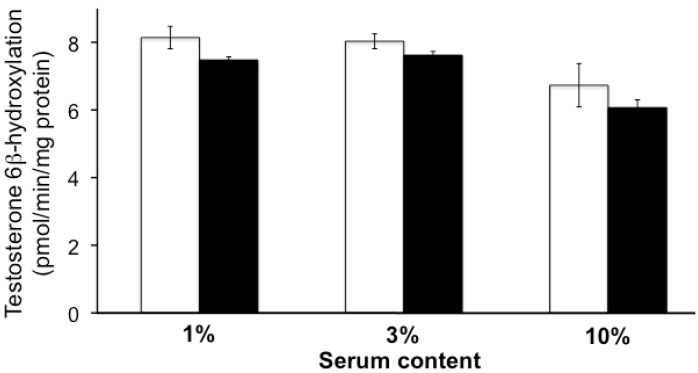
The effects of uremic serum on testosterone 6β-hydroxylation in human liver microsomes. The reaction was performed in human liver microsomes (0.1 mg protein/mL) with a nicotinamide adenine dinucleotide phosphate (NADPH)-generating system and testosterone (50 µM) at 37 °C for 20 min in the presence of normal (NS: open column) or uremic (US: closed column) serum (1%, 3%, 10%). Each column represents the mean ± S.E. of 3 or 4 determinations.

**Figure 2:**
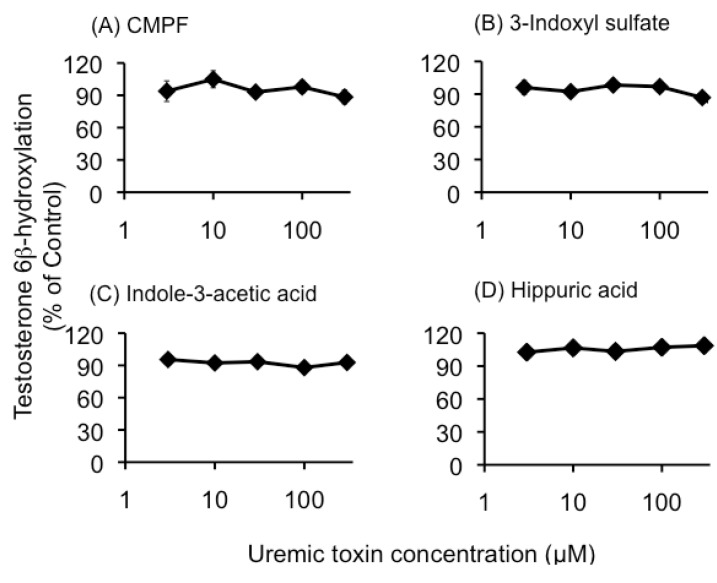
The effects of uremic toxins on testosterone 6β-hydroxylation in human liver microsomes. The reaction was performed in human liver microsomes (0.1 mg protein/mL) with a nicotinamide adenine dinucleotide phosphate (NADPH)-generating system and testosterone (50 µM) at 37 °C for 20 min in the absence or presence of CMPF (**A**), 3-Indoxyl sulfate (**B**), Indole-3-acetic acid (**C**), and Hippuric acid (**D**) (3, 10, 30, 100, and 300 µM). Each column represents the mean ± S.E. of 3 or 4 determinations. These controls were 8.83–10.85 pmol/min/mg protein.

Neither uremic serum nor the four individual uremic toxins (CMPF, 3-indoxyl sulfate, indole-3-acetic acid, and hippuric acid) influenced testosterone 6β-hydroxylation at the concentrations tested in this study (Figure 1 and Figure 2).

### 2.2. CYP3A4 Activity in LS180 Cells Exposed to Uremic Serum or Uremic Toxins

**Figure 3:**
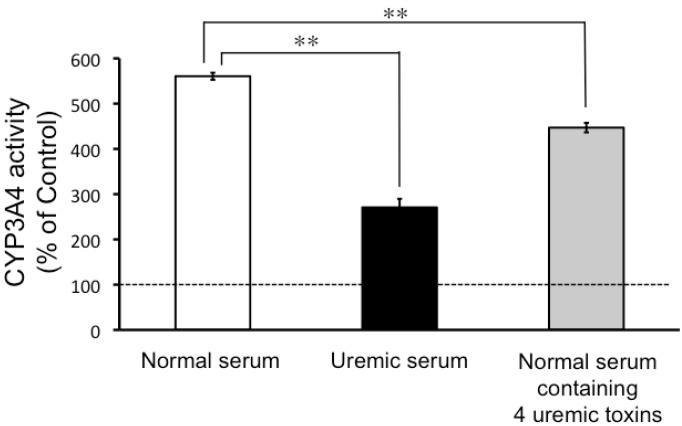
The effects of uremic serum and uremic toxins on CYP3A4 activity in LS180 cells. LS180 cells were seeded at 5 × 10^4^ cells/mL in 24-well plates and cultivated for 4 days. After that, cells were incubated normal serum, uremic serum, or normal serum containing 4 uremic toxins (3-carboxy-4-methyl-5-propyl-2-furanpropanoic acid (CMPF), 3-indoxyl sulfate, indole-3-acetic acid, and hippuric acid) for 24 h. Each point represents the mean ± S.E. (*n* = 4) of P450-GloTMAssays in LS180 cells. Significant differences between the mean values were determined by analysis of variance (ANOVA) followed by Tukey-Kramer test (** *p* < 0.01). CYP3A4: cytochrome P450 3A4.

As can be seen in Figure 3, CYP3A4 activity increased in the LS180 cells treated with normal serum. This effect was reduced in the LS180 cells treated with either uremic serum, or normal serum spiked with uremic toxins (Figure 3).

**Figure 4:**
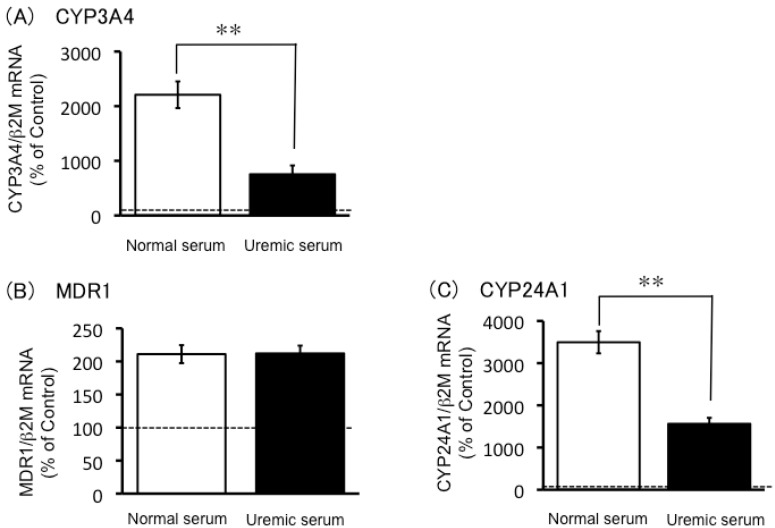
The effects of uremic serum on CYP3A4, MDR1, and CYP24A1 mRNA expression levels in LS180 cells. LS180 cells were seeded at 5 × 10^5^ cells/5 mL into 60 mm dishes and cultivated for 4 days. After that, the cells were treated with normal serum or uremic serum. Cytochrome P450 3A4 (CYP3A4) (**A**), multidrug resistance protein 1 (MDR1) (**B**), and CYP24A1 (**C**) mRNA expression levels were quantified by real-time reverse transcriptase (RT)-PCR. Each point represents the mean ± S.E. (*n* = 3). Significant differences between normal serum and uremic serum were determined by unpaired Student’s t-test (** *p* < 0.01).

### 2.3. Expression of CYP3A4, MDR1 and CYP24A1 mRNA in Uremic Serum-Treated LS180 Cells

The LS180 cells treated with normal serum showed large increases in CYP3A4 and CYP24A1 mRNA levels, and this response was significantly reduced in the uremic serum-treated LS180 cells (Figure 4). Both normal and uremic serum induced MDR1 mRNA levels to a similar extent in LS180 cells (Figure 4B).

### 2.4. Effects of 1,25-Dihydroxyvitamin D and Uremic Toxins on Induction of CYP3A4 mRNA Expression

**Figure 5:**
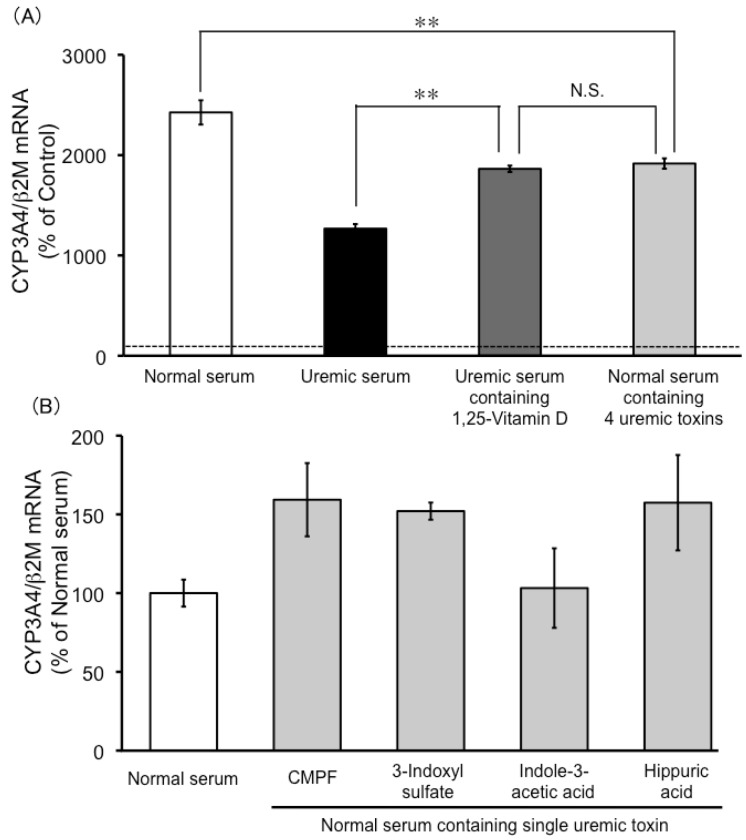
The effects of 1,25-dihydroxyvitamin D and uremic toxins on CYP3A4 mRNA expression levels. LS180 cells were seeded at 5 × 10^5^ cells/5 mL in 60-mm dishes and cultivated for 4 days. The cells were then treated with normal serum, uremic serum, uremic serum containing 1,25-dihydroxyvitamin D, normal serum containing 4 uremic toxins (3-carboxy-4-methyl-5-propyl-2-furanpropanoic acid (CMPF), 3-indoxyl sulfate, indole-3-acetic acid, and hippuric acid) (**A**) or normal serum containing single uremic toxin (**B**). Cytochrome P450 3A4 (CYP3A4) mRNA expression levels were quantified by real-time RT-PCR. Each point represents the mean ± S.E. (*n* = 3). Significant differences between the mean values were determined by analysis of variance (ANOVA) followed by Tukey-Kramer test (** *p* < 0.01, N.S.: not significant).

The concentration of 1,25-dihydroxyvitamin D in uremic serum was significantly lower than that found in normal serum (9.3 ± 1.9 and 62.5 ± 5.0 pmol/L (mean ± SEM), respectively). The effect of uremic serum with added 1,25-dihydroxyvitamin D (50 pmol/L) on CYP3A4 mRNA levels in LS180 cells was investigated. This was found to mitigate the reduced induction observed with uremic serum (Figure 5A), and the effect was statistically significant, but did not restore the response completely to the level seen with normal serum. Furthermore, normal serum with the four added uremic toxins showed significantly suppressed induction of CYP3A4 mRNA expression in LS180 cells (Figure 5A), but normal serum with added single uremic toxin did not (Figure 5B).

## 3. Discussion

The results of our study suggested that neither uremic serum nor uremic toxins directly inhibited CYP3A4 enzyme activity, which was evaluated by measurement of testosterone 6β-hydroxylation in HLMs (Figure 1 and Figure 2). However, the data did indicate that CYP3A4 activity in the LS180 cells was significantly inhibited by uremic serum, and by normal serum spiked with uremic toxins found in uremic serum (Figure 3). Induction of CYP3A4 mRNA expression following exposure of LS180 cells to normal serum was also reduced in the presence of uremic serum (Figure 4A). These results were consistent with those of a report on reduced liver Cyp3a2 mRNA and protein levels in rats with chronic renal failure, compared to normal rats [10]. Therefore, our study suggested that uremic serum downregulated CYP3A4 expression levels and this may contribute to the decreased non-renal clearance mechanisms in individuals with ESRD.

In this study, indoxyl sulfate less than 300 µM did not inhibit CYP3A4 activity (Figure 2), but indoxyl sulfate of higher concentration (1 mM) inhibited CYP3A4 activity such as the report by Sun *et al.* [4]. These results suggest the inhibition of CYP3A4 activity by indoxyl sulfate is minor in clinical situations, because unbound concentration of indoxyl sulfate is about 20 µM (Table 1). 

**Table 1:** Concentration of uremic toxins contained in uremic serum.

Uremic toxin	Serum concentration (μM)		Unbound fraction (%)
	Total	Unbound	
CMPF	179.1 ± 6.9	Not detected	Not calculation
3-Indoxyl sulfate	134.6 ± 0.7	20.2 ± 0.2	14
Indole-3-acetic acid	9.2 ± 0.5	1.7 ± 0.04	18.5
Hippuric acid	238.5 ± 2.1	131.4 ± 0.2	55.5

Values represent the mean ± S.E.

The decreased CYP3A4 mRNA levels may reflect a functional decrease in PXR and/or VDR, which regulate CYP3A4 mRNA expression. However, the present study showed that mRNA levels of another PXR target gene, *MDR1* [11], were comparable in the LS180 cells exposed to uremic serum and to normal serum, indicating that PXR was not involved in the reduced induction of CYP3A4 mRNA expression by uremic serum. On the other hand, the present study found that mRNA expression levels of CYP24A1, a VDR target gene [12], was lower in the uremic serum-treated LS180 cells than in the cells exposed to normal serum. This result indicated that VDR may be involved in the reduced induction of CYP3A4 mRNA expression in the presence of uremic serum.

In ESRD patients, the serum concentration of 1,25-dihydroxyvitamin D is lower than normal subjects [13]. In fact, the present study found that uremic serum contained 15% of the 1,25-dihydroxyvitamin D found in normal serum. Addition of 1,25-dihydroxyvitamin D to uremic serum, to make its final concentration comparable to that in normal serum, significantly mitigated the CYP3A4 induction-suppressing effects (Figure 5A). This finding indicated that the decreased serum 1,25-dihydroxyvitamin D level in ESRD patients could contribute to the decrease in non-renal clearance of CYP3A4 substrates.

Induction of CYP3A4 activity in LS180 cells was also reduced following treatment with normal serum with four uremic toxins added to it, at final concentrations comparable to their serum-unbound concentrations in uremic serum (Figure 3). Furthermore, the induction of CYP3A4 mRNA expression by normal serum was also suppressed on addition of the four uremic toxins to the serum (Figure 5). This finding suggested that the accumulation of uremic toxins in ESRD patients also contributes to their decreased non-renal clearance of CYP3A4 substrates. This hypothesis is supported by the finding that VDR binding to the nuclear VDR-binding element was reduced in the intestines of ESRD rats, and human uremic serum also inhibited the binding of VDR to VDR-binding element in the normal rat intestine [14]. Taken together, these data suggest that VDR malfunction in ESRD patients involves two or more factors, including reduced 1,25-dihydroxyvitamin D concentration and uremic toxin accumulation.

In this study, the mixture of four uremic toxin repressed CYP3A4 mRNA expression, but a single uremic toxin did not significantly affect CYP3A4 mRNA (Figure 5B). Therefore, the influence of each uremic toxin is minor on CYP3A4 mRNA expression, and uremic toxins might have an additive or synergistic effect. Further research will be needed to clarify the cooperative action of uremic toxin.

Other mechanisms, besides CYP3A4 malfunction, can be considered as a cause of delayed elimination of erythromycin. Hepatic clearance of erythromycin involves the organic anion-transporting polypeptide family (OATP) [15]. In addition, CMPF inhibited uptake of erythromycin into rat hepatocytes [4] and digoxin inhibited uptake into human and rat hepatocytes [16]. Some uremic toxins also inhibited erythromycin uptake into OATP-overexpressing cells [17]. Therefore, inhibition of OATP by uremic toxins may also play an important role in the decreased non-renal clearance of erythromycin. It has been reported that although the metabolic clearance of atorvastatin—a substrate of CYP3A4 and OATP—is lower in ESRD patients than in healthy subjects [18], the concentration of midazolam—a substrate of CYP3A4 but not OATP—is comparable in ESRD patients and in healthy subjects [19]. Therefore, the inhibition of OATP may be the primary reason for the altered erythromycin non-renal clearance in ESRD, and the inhibition of CYP3A4 may decrease the clearance synergistically.

## 4. Materials and Methods

### 4.1. Materials

Pooled HLMs were purchased from BD Biosciences (Woburn, MA, USA). Nicotinamide adenine dinucleotide phosphate (NADP^+^), glucose-6-phosphate (G6P), glucose-6-phosphate dehydrogenase (G6PDH), testosterone, carbamazepine, and ketoconazole were purchased from Wako Pure Chemical Industries, Ltd. (Osaka, Japan). Testosterone 6β, 3-indoxyl sulfate and hippuric acid were purchased from Sigma-Aldrich Chemical Co. (St. Louis, MO, USA). Indole-3-acetic acid was purchased from Nacalai Tesque, Inc. (Kyoto, Japan). CMPF was purchased from Cayman Chemical (Ann Arbor, MI, USA). 1,25-Dihydroxyvitamin D was purchased from Enzo Life Sciences (Plymouth Meeting, PA, USA). All other chemicals were reagent- or high-performance liquid chromatography (HPLC)-grade commercial products.

### 4.2. Sera

Human normal serum was purchased from Merck Millipore Co., (Bellerica, MA, USA). Human uremic serum was obtained in a non-invasive manner by collecting spare serum from biochemical tests for ESRD patients (more than 400 patients) who had been receiving hemodialysis at the Shirasagi Hospital (Osaka, Japan). The Ethics Community of Shirasagi Hospital and Kyoto Pharmaceutical University (Kyoto, Japan) approved this study.

### 4.3. Analysis of Uremic Toxins in Uremic Serum

To determine the total serum concentration of uremic toxins, the sample was deproteinized by the addition of 5 volumes of methanol, and filtration by centrifugation with Amicon Centrifree^®^ YM-30 (Millipore, New England, MA, USA) at 1630 × *g* for 10 min. To determine the serum-unbound concentration of uremic toxins in serum, a sample was filtered by centrifugation with YM-30 without prior deproteinization.

Uremic toxin concentration was determined by HPLC with a UV detector by using naphthalene sulfate as an internal standard. The flow rate of the mobile phase was 1.0 mL/min, and the column effluent was monitored at 254 nm. Separation was achieved using a reversed-phase column (4.0 × 250 mm; Inertsil ODS-3; GL Sciences, Inc., Tokyo, Japan). The mobile phase was 50 mM citric acid buffer (pH 4.0)/acetonitrile (95:5 for hippuric acid, and 3-indoxyl sulfate), or 50 mM citric acid buffer (pH 4.0)/methanol (50:50 for CMPF or 70:30 for indoxyl-3-acetic acid). Concentrations of uremic toxins contained in uremic serum were shown in Table 1.

### 4.4. Detection of CYP3A4 Activity Using Testosterone 6β-Hydroxylation

CYP3A4 activity was measured using the method reported by Nishikawa *et al.*, with slight modifications [20]. The reaction mixture contained 0.6 mM NADP^+^, 6 mM G6P, 2 U/mL G6PDH, and 50 µM testosterone in the presence of serum (normal or uremic) or other candidate compounds (0.5% DMSO as control, hippuric acid, indole-3-acetic acid, CMPF, or 3-indoxyl sulfate) in 100 mM sodium phosphate buffer (pH 7.4) with 5 mM MgCl_2_. The reaction mixture was incubated at 37 °C for 5 min prior to the start of the reaction by addition of 0.1 mg protein/mL pooled HLMs. The reaction mixture was incubated at 37 °C for 20 min, and then the reaction was terminated by adding 5 M phosphoric acid.

The assay of 6β-hydroxytestosterone was conducted using an HPLC-UV method reported by Nishikawa *et al.*, with slight modifications [20]. The flow rate of the mobile phase was 1.0 mL/min, and the column effluent was monitored at 245 nm. Separation was achieved using a reversed-phase column (4.0 × 250 mm; Inertsil ODS-3; GL Sciences, Inc., Tokyo, Japan). The mobile phase was 50 mM sodium phosphate buffer (pH 6.0)/methanol/acetonitrile (50:46:4).

### 4.5. Detection of CYP3A4 Activity in LS180 Cells by Using the P450-Glo^TM^ Assays Kit

LS180 cells were seeded at 5 × 10^4^ cells/mL into 24 well plates (Corning Inc., NY, USA), incubated with Dulbecco’s modified Eagle’s medium (D-MEM) containing 10% fetal bovine serum (FBS) for 4 days, and then incubated with the test sera (normal serum, uremic serum, or normal serum spiked with 4 uremic toxins) in FBS-free D-MEM for 24 h. The concentrations of the 4 uremic toxins investigated (2 µM CMPF, 180 µM hippuric acid, 3 µM indole-3-acetic acid, 20 µM 3-indoxyl sulfate) corresponded to their serum-unbound concentrations in uremic serum. CYP3A4 activity in the test serum-treated LS180 cells was detected using P450-Glo™ Assays (Promega, Madison, WI, USA) according to the manufacturer’s instructions. Luminescence intensity was measured using a microplate reader (GENios, Tecan, Seestrasse, Switzerland). The CellQuanti-Blue™ test (BioAssay Systems, Hayward, CA, USA) was used to correct for live cells and the signal quantified using a microplate reader (GENios; excitation, 535 nm; emission 590 nm).

### 4.6. Quantification of mRNA by Real-Time Reverse Transcription-Polymerase Chain Reaction Analysis

LS180 cells (5 × 10^5^ cells) were seeded on plastic culture dishes (60 mm in diameter) and incubated with 5 mL D-MEM with 10% FBS for 4 days. The culture medium was then exchanged with the test sera (normal serum, uremic serum, uremic serum containing 50 pmol/L 1,25-dihydroxyvitamin D, or normal serum spiked with 4 uremic toxins) in FBS-free D-MEM for 24 h. The final concentrations of the 4 uremic toxins (18 µM CMPF, 25 µM hippuric acid, 1 µM indole-3-acetic acid, and 15 µM 3-indoxyl sulfate) corresponded to their concentrations in 10% uremic serum.

Total RNA was extracted from the cells by using the GenElute™ Mammalian Total RNA Miniprep Kit (Sigma-Aldrich). Reverse transcriptase (RT) reactions were performed using the ReverTra Ace^®^ qPCR RT Kit (Toyobo, Osaka, Japan) in a thermal cycler (i-Cycler; Bio-Rad, Hercules, CA, USA). Real-time PCR was performed using THUNDERBIRD™ SYBR^®^ qPCR Mix (Toyobo) in a 7500 Real-Time PCR System (Applied Biosystems, Carlsbad, CA, USA). The sequences of the primers were as follows: CYP3A4 forward, 5'-GCC TGG TGC TCC TCT ATC TA-3' and reverse, 5'-GGC TGT TGA CCA TCA TAA AAG-3'; multidrug resistance protein 1 (MDR1) forward, 5'-GTG GTG GGA ACT TTG GCG G-3' and reverse, 5'-TAC CTG GTC ATG TCT TCC TCC-3'; CYP24A1 forward, 5'-CAA ACC GTG GAA GGC CTA TC-3' and reverse, 5'-CAG TCT TCC CCT TCC AGG ATC A-3'; β2-microgloblin (β2M) forward, 5'-TGC TCG CGC TAC TCT CTC TTT C-3' and reverse, 5'-TTC TCT GCT TGA CGT GAG TAA-3'.

In this study, we confirmed that uremic serum and uremic toxins did not affect β2M mRNA expression.

### 4.7. Quantification of 1,25-Dihydroxyvitamin D

Serum levels of 1,25-dihydroxyvitamin D were measured using the 1,25-(OH)_2_-Vitamin D ELISA Kit (Immundiagnostik AG, Bensheim, Germany) according to the manufacturer’s instructions. Absorbance was measured using a microplate reader (GENios; measurement wavelength, 450 nm; reference wavelength, 620 nm).

### 4.8. Data Analysis

All data are presented as mean ± standard error of the mean. Significance of any differences between means were determined using Student’s *t* test or analysis of variance (ANOVA) followed by Dunnet test or Tukey-Kramer test. A *p* value of less than 0.05 was considered statistically significant.

## 5. Conclusions

In conclusion, the present study indicated that decreased levels of 1,25-dihydroxyvitamin D and accumulation of uremic toxins might contribute to the decreased non-renal clearance of CYP3A4 substrates, such as erythromycin and atorvastatin, in ESRD. Further studies might be able to identify the uremic toxins involved in VDR dysfunction.
